# Facile preparation of YAG:Ce nanoparticles by laser irradiation in water and their optical properties

**DOI:** 10.1186/s40064-016-1958-2

**Published:** 2016-03-15

**Authors:** Noriyuki Tsuruoka, Takao Sasagawa, Tokuo Yodo, Mamoru Yoshimoto, Osamu Odawara, Hiroyuki Wada

**Affiliations:** Interdisciplinary Graduate School of Science and Engineering, Tokyo Institute of Technology, 4259 Nagatsuta-cho, #J2-41, Midori-ku, Yokohama, 226-8502 Japan; Materials and Structures Laboratory, Tokyo Institute of Technology, 4259 Nagatsuta-cho, Midori-ku, Yokohama, 226-8503 Japan; Graduate School of Engineering, Osaka Institute of Technology, 5-16-1 Ohmiya, Asahi-ku, Osaka, 535-8585 Japan

**Keywords:** Nanoparticle, Laser ablation in liquid, Yellow phosphor, YAG:Ce

## Abstract

Yttrium aluminum oxide Y_3_Al_5_O_12_ (YAG:Ce) nanoparticles were prepared by laser ablation in liquid, and the photoluminescence (PL) properties of the nanoparticles were investigated. A pellet of YAG:Ce synthesized by co-precipitation in deionized water was irradiated with a focused laser beam to obtain a solution containing dispersed nanoparticles. The compositions and morphologies of the nanoparticles were investigated by X-ray diffraction, transmission electron microscopy, scanning electron microscopy, energy dispersive X-ray analysis, and dynamic light scattering. PL and photoluminescence excitation (PLE) spectra at room temperature and low temperature were measured using a fluorescence spectrophotometer. Nanoparticles of YAG single phase as a matrix were obtained by irradiation at high laser energy density. The average particle size was approximately 9 nm, although the nanoparticles were slightly aggregated. The broad peak centered at 540 nm in the PL spectrum was asymmetrically broadened at shorter wavelength. The intensity of the PLE peak centered at 340 nm decreased with increasing energy density of the laser beam. These phenomena were related to the nanosize effect of the YAG:Ce phosphor.

## Background

White light-emitting diodes (LEDs) consist of yellow phosphor Y_3_Al_5_O_12_ (YAG:Ce) and a blue LED, for which the Nobel Prize in Physics was awarded in 2014. White LEDs are used instead of light bulbs because they save energy and have longer lifetimes. Because the yellow phosphor is placed in front of the blue LED, the yellow phosphor partially absorbs blue light and emits yellow light. Part of the blue light from the blue LED penetrates the yellow phosphor, and the yellow and blue lights create quasi-white light in human eyes. Scattering loss, which is proportional to the 6th power of particle size, reduces the intensity of blue light. Therefore, many studies on reducing the particle size of phosphors have recently been reported (Kasuya et al. 2007; Wang et al. 2015). Nanosized phosphors are very useful in various fields of research such as biomedicine and energy (Ikehata et al. 2015; Kobayashi et al. 2014).

Several methods are available for the production of nanoparticles. One widely used method is a solution method based on chemical reaction. In general, because the process temperature of this method is low, the crystallinity of the prepared nanoparticles is low. If the prepared nanoparticles are annealed in a furnace to increase the crystallinity, the particles are connected to each other by necking, and particle size is increased by crystal growth. Unreacted starting materials occasionally remain in prepared samples as by-products. Laser ablation in liquid is a facile method for the preparation of dispersed nanoparticles (Patil et al. 1987; Neddersen et al. 1993; Fojtik and Henglein 1993; Mafune et al. 2000; Sajti et al. 2010). Nanosized phosphors have been prepared using this method (Wang et al. 2015; Ikehata et al. 2015; Nunokawa et al. 2014). In the case of dielectric inorganic materials, highly crystalline nanoparticles are obtained by this method because highly crystalline bulk materials are transformed into nanoparticles. Pure nanoparticles without by-products can be prepared using this method because chemical reactions are not included in the process. Furthermore, because multi-element nanoparticles can be obtained using this method, it is suitable for the preparation of nanosized phosphors.

In this study, YAG:Ce nanoparticles were prepared by laser ablation in liquid, and their optical properties were investigated. The effect of the energy density of the laser on the prepared particles was elucidated. The influence of particle size reduction on the photoluminescence (PL) properties of the nanoparticles was also investigated.

## Results and discussion

**Fig. 1:**
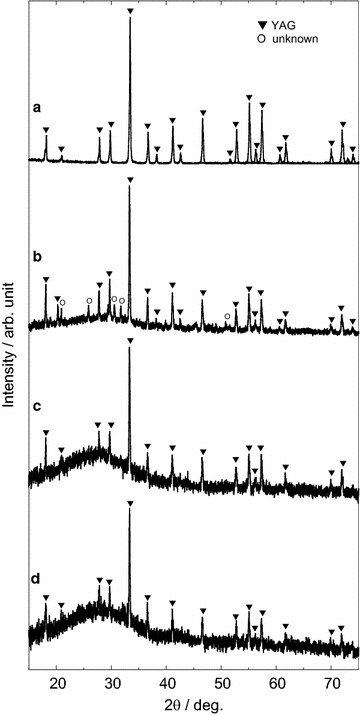
XRD patterns of **a** target, **b** nanoparticle (laser energy density: 1.1 J/cm^2^), **c** nanoparticle (1.8 J/cm^2^), and **d** nanoparticle (3.7 J/cm^2^)

Figure 1b–d show the X-ray diffraction (XRD) patterns of the prepared nanoparticles. For reference, the spectrum of the target is shown in Fig. 1a. The energy densities of laser irradiation used to prepare the particles were (b) 1.1 J/cm^2^, (c) 1.8 J/cm^2^, and (d) 3.7 J/cm^2^. In general, Y_3_Al_5_O_12_ exhibits a garnet structure (A_3_B′_2_B″_3_O_12_, space group is *Ia*3*d*), which includes eight units (Galasso 1970). Three metal sites among 96 oxygen atoms exist; Ion A means 24 dodecahedral sites, Ion B′ means 16 tetrahedral sites, and Ion B″ means 24 octahedral sites in a unit cell (Galasso 1970). Ce ion is substituted at the Y site because the ionic radius of Ce (1.14 Å with coordination number (CN) = 8) is slightly larger than that of Y (1.02 Å with CN = 8) (Jia 1991). Figure 1a indicates that the YAG:Ce target was successfully synthesized without impurities using the sol–gel method. The nanoparticles prepared using a low energy density (1.1 J/cm^2^) contained an unknown impurity (Fig. 1b). However, pure YAG:Ce nanoparticles without metastable phases such as YAlO_3_ (YAP) and Y_4_Al_2_O_9_ (YAM) were prepared by laser ablation in liquid using energy densities higher than 1.8 J/cm^2^ (Fig. 1c, d). Particle size calculated by Scherrer equation of Figs. (a), (b), (c), and (d) were 65, 58, 51, and 56 nm, respectively.

**Fig. 2:**
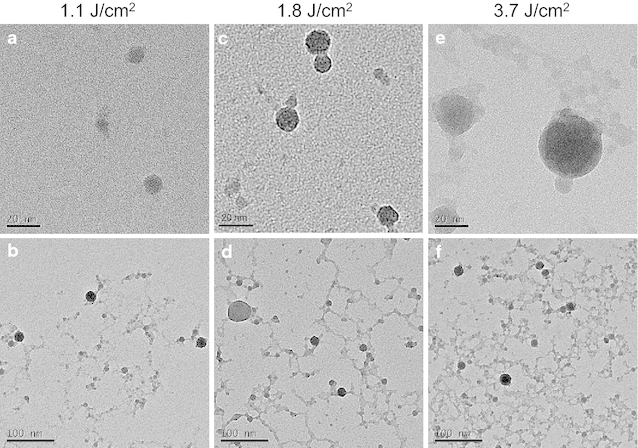
TEM images of prepared nanoparticles prepared at each laser energy density (laser energy densities: **a**, **b** 1.1 J/cm^2^; **c**, **d** 1.8 J/cm^2^; and **e**, **f** 3.7 J/cm^2^. Magnifications: **a**, **c**, **e** low and **b**, **d**, **f** high)

**Fig. 3:**
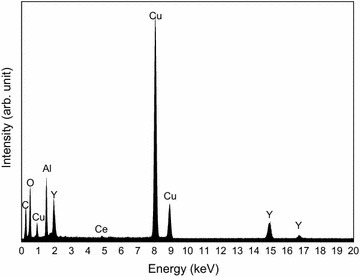
EDX spectra of nanoparticles (laser energy densities: 3.7 J/cm^2^)

**Fig. 4:**
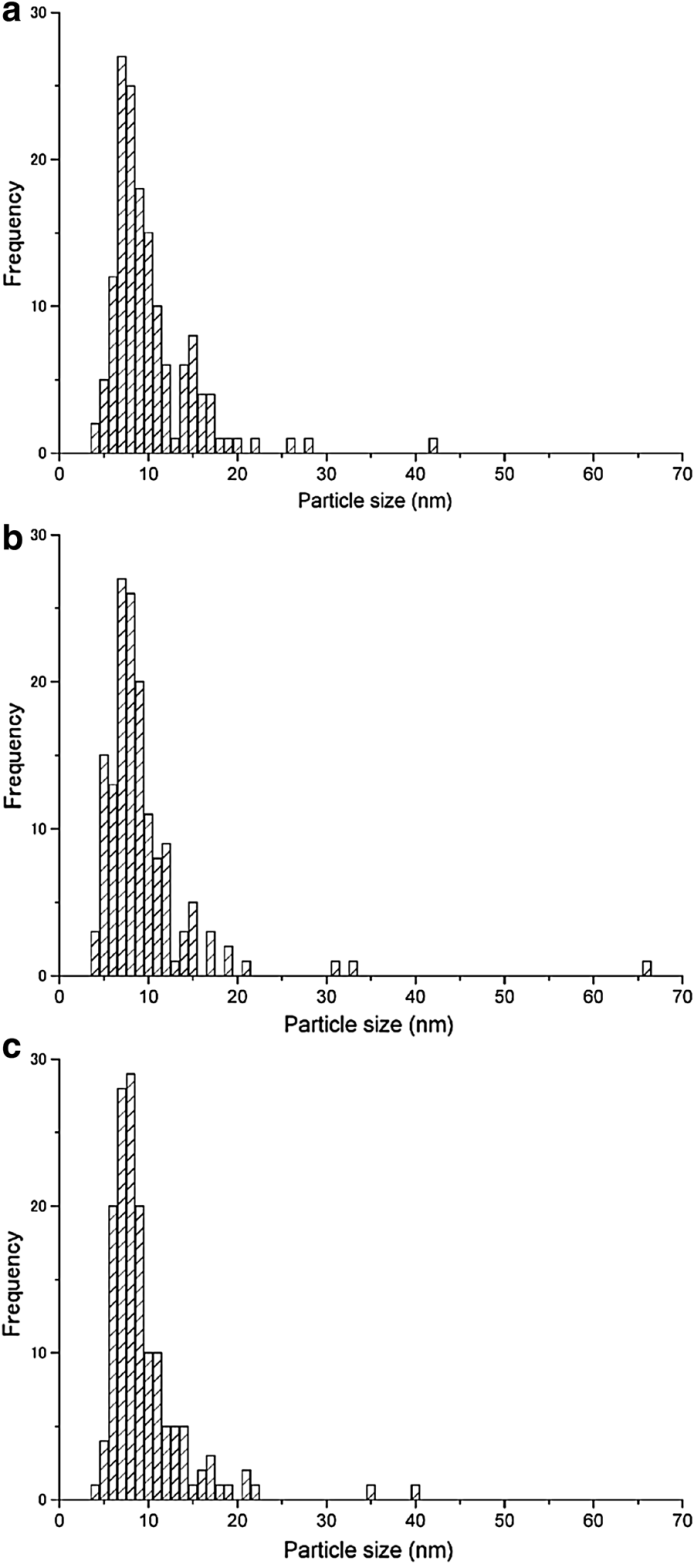
Primary particle size distributions of nanoparticles measured by TEM (laser energy densities: **a** 1.1 J/cm^2^, **b** 1.8 J/cm^2^, and **c** 3.7 J/cm^2^)

**Fig. 5:**
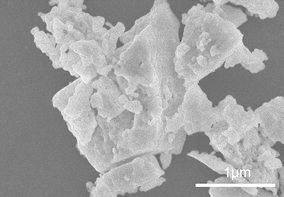
SEM images of YAG:Ce target

Figure 2 shows the transmission electron microscopy (TEM) images of the nanoparticles prepared at each laser energy density. The energy-dispersive X-ray analysis (EDX) results for the nanoparticles (3.7 J/cm^2^) are shown in Fig. 3. The observed peaks are the elements of YAG:Ce, suggesting the formation of YAG:Ce nanoparticles, although peaks corresponding to the copper TEM grid and the elastic carbon membrane are also observed. The composition (atomic percentages) of the nanoparticles (Table 1) was calculated on the basis of the EDX peaks. The atomic percentage of Al was slightly lower than that of YAG, and that of Y was slightly higher than that of YAG. This result has two possible explanations: (1) the evaporation of some elements by the high temperature during laser ablation; and (2) the existence of minor amounts of metastable phases such as YAP and YAM. Two kinds of nanoparticles are observed in Fig. 2: tiny nanoparticles with sizes of approximately 10 nm and spherical nanoparticles with sizes of several tens of nanometers. The former particles were connected to each other. These structures have been frequently observed in nanoparticles prepared by laser ablation in liquid (Inasawa et al. 2005; Nunokawa et al. 2012). Tiny nanoparticles prepared by laser ablation in liquid aggregate; thus, they would be melted by laser beam irradiation to form these structures. The larger nanoparticles are formed by laser melting in liquid (Ishikawa et al. 2007, 2010; Kawasoe et al. 2015). The primary particle size distribution of the nanoparticles at each energy density is shown in Fig. 4. The average particle size (10 nm) was not changed by the laser energy density under the experimental conditions. Although the sizes of nanoparticles were slightly smaller than those of XRD measurement, these sizes was the same in the order of magnitude. Particle of target measured by scanning electron microscopy (SEM) was shown in Fig. 5. The particle size of target in Fig. 5 was larger than that calculated by Scherrer equation because target prepared by co-precipitation method would consist of fine crystallites as poly crystal. In a previous study, tiny and coarse nanoparticles with particle sizes of several hundreds of nanometers were prepared simultaneously (Nunokawa et al. 2012). The target of laser ablation in liquid was the aggregation of ceramic grains. The coarse nanoparticles consisted of the aggregation of ceramic grains. The primary particle size of the tiny nanoparticle was much smaller than the size of the ceramic grains. Therefore, the coarse nanoparticles would be formed by the partial fragmentation of the aggregation of ceramic grains of the target, while the tiny nanoparticles would be formed by the division of the ceramic grains. If the bonding force between the ceramic grains was weak, coarse nanoparticles would be generated by laser irradiation. In this study, almost all of the fabricated nanoparticles were tiny nanoparticles. Coarse nanoparticles were not generated because the bonding force between the ceramic grains would be greater than a force related to laser irradiation, such as a shock wave caused by high sintering temperature. And the amount of nanoparticles would be increased with increase in ablation time as shown in our previous study (Kobayashi et al. 2013).

**Table 1:** Atomic percentages of the nanoparticles (energy densities: 3.7 J/cm^2^)

Element	Atomic percent (at.%)
O (K)	59
Al (K)	21
Ce (L)	1
Y (K)	19
Total	100

**Fig. 6:**
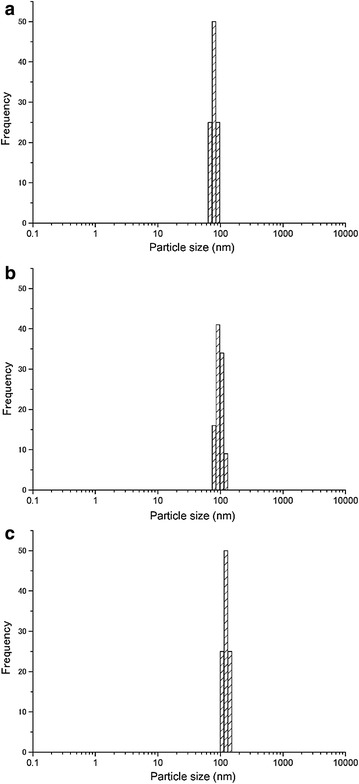
Secondary particle size distributions of nanoparticles measured by DLS (laser energy densities: **a** 1.1 J/cm^2^, **b** 1.8 J/cm^2^, and **c** 3.7 J/cm^2^)

The secondary particle size distribution of the nanoparticles at each laser energy density was measured by dynamic light scattering (DLS), as shown in Fig. 6. The secondary particles were approximately 100 nm, although this value increased slightly with increasing laser energy density. Because the primary nanoparticle size was 9 nm, nanoparticle aggregation occurred, as observed in the low-magnification TEM images. The spherical hydrodynamic diameter measured by DLS was not exactly the same as true diameter of aggregation obtained from the TEM images because of string-like nanoparticles.

**Fig. 7:**
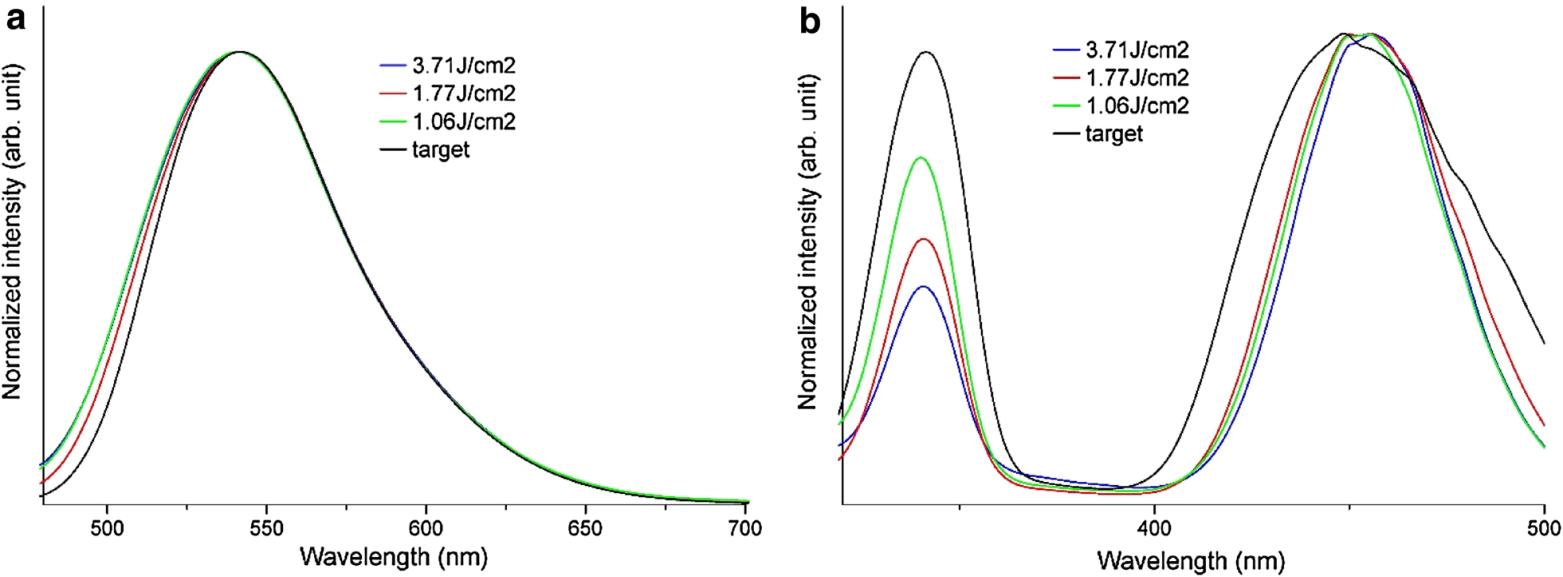
**a** PL and **b** PLE spectra of nanoparticles of target and nanoparticles at each laser energy density (*λ*_EX_ = 445 nm; *λ*_EM_ = 540 nm)

Figure 7a, b show the PL and photoluminescence excitation (PLE) spectra of the nanoparticles, respectively. The PL spectrum (Fig. 7a) exhibits a broad peak centered at about 540 nm, a typical emission of YAG:Ce. This peak is broadened asymmetrically in the short-wavelength region. A similar tendency was observed in a previous study because of a change in the crystal field strength around Ce^3+^ due to the nanosized effect (Kasuya et al. 2007). The PLE spectra (Fig. 7b) exhibits two broad peaks centered at approximately 340 and 450 nm, corresponding to the transitions from the 4*f*-orbital (^2^*F*_5/2_; lowest energy level) to the 5*d*-orbital [^2^*B*_1g_ (higher-energy level) and ^2^*A*_1g_ (lower-energy level)], which are split by the crystal field strength (Lu et al. 2002). These peaks were drastically changed by nanosizing. The peak around 340 nm was blue-shifted by 2 nm, and the peak around 450 nm was red-shifted by 2 nm and narrowed. The intensity of the peak around 340 in the PLE spectrum was reduced with increasing laser energy density (Bachmann et al. 2009). This may be attributed to low transition probability and non-radiative relaxation by defects. Another possible reasons is the saturation of peak intensity around 450 nm as shown in the following (Bachmann et al. 2009). The peak intensities of Ce^3+^ in the PLE spectrum increase with increasing Ce^3+^ concentration and are likely to be saturated. The peak intensity around 450 nm is more likely to be saturated than that around 340 nm. Therefore, the decrease in Ce^3+^ concentration reduces the saturated peak intensity around 340 nm and then reduces that around 340 nm. These phenomena were observed in Fig. 7b. Two possible reasons for the decrease in Ce^3+^ concentration observed in this study are as follows: (1) the decrease in concentration occurs because of the high temperature caused by laser irradiation; and (2) the oxidation of Ce^3+^ to Ce^4+^ occurs during the high-temperature process.

**Fig. 8:**
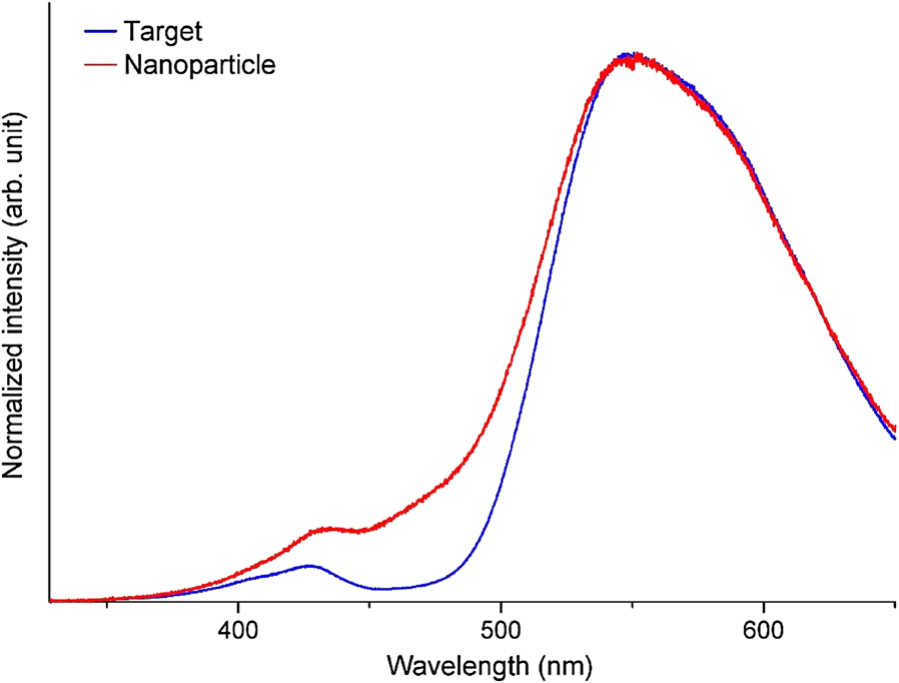
Low-temperature PL spectra of the target and nanoparticles (laser energy density: 1.1 J/cm^2^) at 14.4 K

Figure 8 shows the low-temperature PL spectra of the target and nanoparticles, which clearly indicate the asymmetric broadening of the emission peak at short wavelength. This broadening would be attributed to the decrease in the crystal field strength around Ce^3+^ due to the nanosized effect. A red shift in the peak around 430 nm was observed simultaneously. This phenomenon would indicate the slight stabilization of the higher 5*d* energy level (^2^*B*_1g_), implying that the energy level was decreased. This would be also attributed to the decrease in crystal field strength around Ce^3+^ due to the nanosized effect.

## Conclusions

YAG:Ce nanoparticles were successfully prepared by laser ablation in liquid when the laser energy density was sufficiently high. The atomic percentages of the nanoparticles were slightly changed by laser ablation. The average primary particle size of the nanoparticles was approximately 9 nm, although the nanoparticles aggregated slightly to form a string-like structure. Particle size was not dependent on the laser energy density in this study. The optical properties were significantly changed by the nanosize effect. The intensity of the shorter peak in the PLE spectrum decreased with increasing laser energy density. The peak at longer wavelength in the PLE spectrum was narrowed by the nanosizing effect. The peaks in the PLE spectrum were shifted due to nanosizing, and the broad peak in the PL spectrum was asymmetrically broadened.

## Methods

Y(NO_3_)_3_·6H_2_O (99.99 %), Al(NO_3_)_3_·9H_2_O (>98 %), Ce(NO_3_)_3_·6H_2_O (99.9 %), ethanol (99.5 %), and ammonia water (Kanto Chemical) were used without further purification. YAG:Ce powder was synthesized by co-precipitation. Yttrium nitrate, aluminum nitrate, and cerium nitrate were ultrasonically dissolved in DI water (25 ml). Ammonia (5 M) was added to this solution to adjust the pH to 8, and this solution was aged for 15 min for precipitation to occur. The precipitate was washed three times by centrifugation (5000 rpm, 10 min), dried in an oven (60 °C, 12 h), and calcined in an electric furnace (1000 °C, 1 h) to obtain YAG:Ce powder. The cylindrically pressed powder ($\phi$ 9 mm, 0.80 g, 480 MPa, 10 min) was sintered (1500 °C, 1 h) under reducing atmosphere (H_2_ 5 %/Ar 95 %) to prevent the oxidation of Ce^3+^ to Ce^4+^. In general, high-temperature sintering is important because metastable phases such as YAP and YAM are synthesized as by-products at <1500 °C.

YAG:Ce nanoparticles were obtained by laser ablation in liquid. The above YAG:Ce pellet was immersed in DI water (3 ml) in a cuvette and irradiated with a focused pulse laser beam [Nd:YAG/second harmonic generation (SHG), wavelength = 532 nm, pulse duration = 13 ns, repetition rate = 10 Hz] for 10 min. The energy density of the laser was varied. The particles obtained in the supernatant of the solution were characterized by the following methods.

XRD (PANalytical X’pert-PRO-MPD; X-ray, CuKα1:CuKα2 = 2:1; 40 V; 30 mA; divergence slit = 0.19 mm; receiving slit = 0.1 mm; scan step = 0.02°; scan speed = 1.85°/min) was used to identify the nanoparticles. The morphology and composition of the nanoparticles and target were investigated by TEM (JEOL JEM-2010F, acceleration voltage = 200 kV) and SEM (Hitachi High-Technologies S-4800, acceleration voltage = 5 kV) with EDX. The secondary particle size of the nanoparticles in solvent was measured by DLS (Marvern Instruments, Zetasizer Nano ZS; laser wavelength = 633 nm; refractive index of sample = 1.80; refractive index of solvent = 1.330; viscosity of solvent = 0.8872 cP; temperature = 25 °C; PMMA cell). PL and PLE spectra were measured using a fluorescence spectrophotometer (Hitachi High Technologies F-7000; excitation wavelength = 445 nm; emission wavelength = 540 nm; scan speed = 240 nm/min). The nanoparticles were dried in a vacuum oven to avoid reduction of PL intensity by energy transfer to water molecules. The low-temperature PL spectrum of the nanoparticles (He–Cd laser; excitation wavelength = 325 nm; power = 7.1 mW; temperature = 14.4 K; slit width = 0.02 mm; time constant = 100 ms) was also collected after the dried powder was encapsulated in liquid glass.

## Abbreviations

YAG: yttrium aluminum oxide Y_3_Al_5_O_12_
XRD: X-ray diffraction
TEM: transmission electron microscopy
EDX: energy dispersive X-ray analysis
SEM: scanning electron microscopy
DLS: dynamic light scattering
PL: photoluminescence
PLE: photoluminescence excitation
LED: light-emitting diode
CN: coordination number
YAP: YAlO_3_
YAM: Y_4_Al_2_O_9_
DI: deionized
SHG: second harmonic generation
